# Liver metastasis of a neuroendocrine tumor demonstrates intense uptake in PSMA-PET—but not its lymph-node metastasis and primary-tumor

**DOI:** 10.1007/s00259-023-06120-8

**Published:** 2023-02-13

**Authors:** Erik Winter, Stefanie Zschäbitz, Clemens Kratochwil

**Affiliations:** 1 Department of Nuclear Medicine, University Hospital Heidelberg, INF 400, 69120 Heidelberg, Germany; 2 Department of Medical Oncology, University Hospital Heidelberg, National Center of Tumor Diseases, Heidelberg, Germany

## Image of the month

During the last decade, PSMA-PET/CT became a mainstay in the imaging of prostate cancer. However, PSMA is also expressed in the neo-vasculature of various non-prostatic tumors [1]. Amongst other somatostatin receptor-targeted tracers, DOTATOC-PET/CT is the current gold standard for imaging of well-differentiated neuroendocrine tumors arising in the gastrointestinal tract [2].

This image presents a patient who was originally diagnosed with a high-grade prostate carcinoma (Gleason score 9, initial PSA 48 ng/ml). Consequently, an atypically located liver lesion was histologically proven to be a well-differentiated neuroendocrine tumor (G2). Due to his transplant kidney, the patient was not allowed to receive contrast media; hence, he received staging with [^18^F]F-PSMA-1007 and [^68^ Ga]Ga-DOTATOC PET/CT.

In the PSMA-PET, the uptake of the prostate carcinoma was SUV_max_ 55.5 and the suspected liver metastasis was SUV_max_ 106.2; no uptake was seen in the neuroendocrine primary tumor and only faint uptake (SUV_max_ 8.5) in a peritoneal lymph node. In the DOTATOC-PET, the uptake of the duodenal neuroendocrine primary tumor was SUV_max_ 67.9, the positive lymph node in the related drainage was SUV_max_ 91.2, and the liver metastasis was SUV_max_ 221.3; the adenocarcinoma of the prostate was somatostatin-receptor negative.

The presented image contains two interesting points. First, it demonstrates the high variability of PSMA expression in non-prostatic tumor lesions and its dependency of the local tumor microenvironment (e.g., site of metastasis, neoangiogenetic activity, organ-specific perfusion effects). Second, it serves as a reminder that an uncritical belief in strong biological signals obtained with highly specific tracers can lead to an overestimate of the “histo-radiological” performance of molecular imaging. A clinically uncommon situation—such as suspicion of liver metastatic prostate cancer without additional lymph node or bone metastases—still needs histopathological validation! 
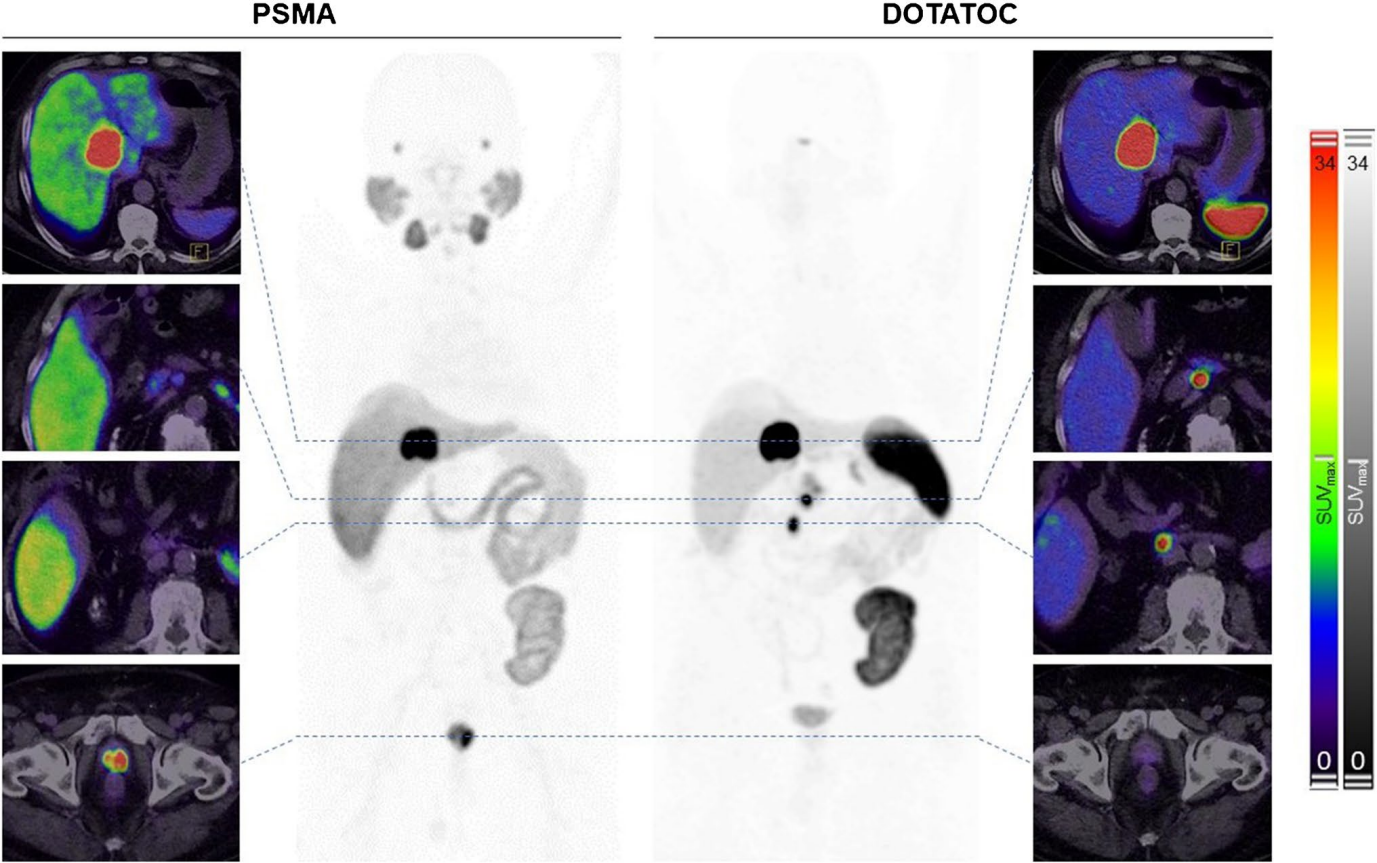


## Data Availability

The data that support the findings of this study are available from the corresponding author, EW, upon reasonable request.
